# Comparing the Profiles of Raw and Cooked Donkey Meat by Metabonomics and Lipidomics Assessment

**DOI:** 10.3389/fnut.2022.851761

**Published:** 2022-03-25

**Authors:** Mengmeng Li, Wei Ren, Wenqiong Chai, Mingxia Zhu, Limin Man, Yandong Zhan, Huaxiu Qin, Mengqi Sun, Jingjing Liu, Demin Zhang, Yonghui Wang, Tianqi Wang, Xiaoyuan Shi, Changfa Wang

**Affiliations:** ^1^College of Agronomy and Agricultural Engineering, Liaocheng Research Institute of Donkey High-Efficiency Breeding and Ecological Feeding, School of Materials Science and Engineering, Liaocheng University, Liaocheng, China; ^2^Shandong Animal Husbandry Station, Jinan, China

**Keywords:** donkey meat, metabonomics, lipidomics, taste compounds, lipid profile

## Abstract

Heat cooking of meat gives it a specific taste and flavor which are favored by many consumers. While the characteristic taste components of chicken, duck, pig, and seafood have been studied, there is a lack of information about the molecular components that give donkey meat its unique taste. Here, the characterization profiles of raw donkey meat (RDM) and cooked donkey meat (CDM) meat by metabonomics and lipidomics. The results showed that a total of 186 metabolites belonging to 8 subclasses were identified in CDM and RDM, including carbohydrates (27.42%), amino acids (17.20%), lipids (13.44%), and nucleotides (9.14%). In total, 37 differential metabolites were identified between CDM and RDM. Among these, maltotriose, L-glutamate, and L-proline might predominantly contribute to the unique umami and sweet taste of donkey meat. Comprehensive biomarker screening detected 9 potential metabolite markers for the discrimination among RDM and CDM, including L-glutamate, gamma-aminobutyric acid, and butane-1, 2, 3, 4-tetrol. Moreover, a total of 992 and 1,022 lipids belonging to 12 subclasses were identified in RDM and CDM, respectively, mainly including triglycerides (TGs) and glycerophospholipids (GPs). Of these lipids, 116 were significantly different between CDM and RDM. The abundances of 61 TGs rich in saturated and monounsaturated fatty acids were retained in CDM, whereas the abundances of 37 GPs rich in polyunsaturated fatty acids were reduced, suggesting that TGs and GPs might be the predominant lipids for binding and generating aroma compounds, respectively. A total of 13 lipids were determined as potential markers for the discrimination among RDM and CDM, including PC(O-16:2/2:0), LPE(22:5/0:0), and PC(P-16:0/2:0). In conclusion, this study provided useful information about the metabolic and lipid profiles of donkey meat which may explain its unique taste and flavor, which could serve as a basis for the development and quality control of donkey meat and its products.

## Introduction

Donkies (*Equus asinus*) are mainly distributed in Africa, Asia, and the Americas, and have played important roles throughout human history, such as instruments for cargo transportation, cultural communication, and agricultural production (1, 2). During the last two decades, donkeys have gradually lost their traditional role as working animals, and are instead mainly used to provide the production of leather (ejiao), milk, meat, and cosmetics (3). In particular, donkey meat and its products have become more and more popular amongst consumers in the past few years due to perceived health benefits, especially in China. Compared to sheep and cow meat, which are generally regarded as more suitable for human consumption, donkey meat is much more tender, and has a higher percentage of protein, essential amino acids and polyunsaturated fatty acids (PUFAs) as well as lower fat, cholesterol and calorie content (4–6).

People are usually more accustomed to eating traditional cooked donkey meat, such as meat rolls and pie made of stewed donkey meat and donkey meat sauce (7). Heat cooking of meat gives it a specific taste and flavor which are favored by Chinese consumers. The taste characteristics of meat products are determined by non-volatile taste components, which mainly include amino acids, small peptides, nucleic acid metabolites, and inorganic salts (8). Previous studies have shown that while the amino acids glutamate, glycine, alanine, valine, and aspartate all contribute to umami taste, glutamate and aspartate are especially important (9). Additionally, nucleotides such as inosine monophosphate and guanosine monophosphate are closely related to umami (10). While the characteristic taste components of chicken, duck, pig meat, and seafood have been studied (11–15), there is a lack of information about the molecular components that give donkey meat its unique taste.

Cooked meat flavor develops during the heating process mainly through chemical reactions involving lipids and low molecular weight compounds (16). Lipids are solvent precursors of aroma compounds such as 1-octen-3-ol and hexanal, which indirectly affect the sensory characteristics of meat (17). Moreover, due to the lipophilic nature of most aroma compounds, lipids are considered to be critical aroma retainers (18, 19). Triglycerides (TGs) and glycerophospholipids (GPs) are two major categories of lipid of meat, and GPs are higher proportion of PUFA in comparison with TGs (20). Furthermore, donkey meat is particularly rich in GPs, which constitute 20–30% of the total lipid content (21). Previous studies have shown that TGs and GPs might be the predominant lipids in meat for binding and generating aroma compounds during cooking, respectively (22). However, it is still poorly understood how cooking changes the lipid composition of donkey meat and how the unique flavor is generated.

Metabolomics and lipidomics have become critical methods to analyze the nutritional profiles of foods (23, 24). Mass spectrometry (MS)-based metabolomics has previously been used to elucidate the metabolic profiles and to identify biomarkers in chicken meat in different diets (25), different meat of breeds and type (26, 27), and meat during storage and processing (28). In addition, MS-based lipidomics has been used for composition analysis, quality identification, origin traceability and authenticity identification of meat (29, 30). A recent study has reported that 1,143 lipids belonging to 14 subclasses were characterized in donkey meat by using MS-based lipidomics, the GP, glycerolipid (GL), and sphingolipid (SP) metabolism were identified as key ways regulating intramuscular fat (21). However, these methods have seldom been used to compare the metabolic and lipid profiles of raw donkey meat (RDM) and cooked donkey meat (CDM). Therefore, the present study aimed to obtain a comprehensive characterization of the metabolic and lipid profiles of RDM and CDM, and to analyze differences in the levels of these molecules between the two states as well as to screen for potential biomarkers. This study provides important information for understanding the unique taste and flavor of donkey meat.

## Materials and Methods

### Chemicals and Materials

Acetonitrile, isopropanol, chloroform, methanol, potassium hydroxide, and boron trifluoride ether were purchased from Thermo Fisher Scientific (Shanghai, China). The 37 component fatty acid methyl esters (FAMEs) mix standards (CRM47885), methoxyamine, and ammonium formate were purchased from Sigma-Aldrich (Shanghai, China). N,O-Bis(trimethylsilyl)trifluoroacetamide with 1% trimethylchlorosilane (BSTFA-TMCS) and formic acid were purchased from TCI Chemical Industry Development Co., Ltd (Shanghai, China). The 0.22 μm polyvinylidene fluoride membrane was purchased from Jin Teng Biological Technology Co., Ltd. (Tianjin, China).

### Sample Collection and Treatment

Fresh biceps femoris tissue samples from six Dezhou donkeys were obtained from Shandong Dong'e Tianlong Food Co., Ltd (Shandong, China) and immediately placed in liquid nitrogen and stored at −80°C for further analysis. Before cooking, meat samples were wrapped in a vacuum foil pouch to avoid the loss of weight, lipids and metabolites during the heat treatment, and then cooked in a water bath at 100°C for 90 min, and stored at −80°C for further analysis.

### Samples Preparation and Metabolite Detection

Muscle samples (50 mg) were ground with 0.5 mL of a mixture of acetonitrile, isopropanol, and water (3:3:2, v/v/v) using a high-flux tissue grinder (Xinzhi Biotechnology Co., Ltd, Ningbo, China), and then sonicated in an ice water bath for 5 min. Another 0.5 mL of the same solution was again added and the samples sonicated for 5 min in ice water bath. The samples were then centrifuged at 10,000 × g for 2 min, and 500 μL of the resultant supernatant was concentrated to dryness under vacuum, and then dissolved in 80 μL of 20 mg/mL methoxyamine pyridine solution, and incubated at 60°C for 60 min. Finally, the 100 μL BSTFA-TMCS (99:1) reagent was added for de derivatization at 70°C for 90 min before the samples were centrifuged at 12,000 × g for 3 min, and 100 μL of supernatant was taken and tested for gas chromatography time-of-flight (GC-TOF) upper detection within 24 h. In order to evaluate the stability and reliability of metabolomics analysis method, a quality control (QC) sample was prepared by pooling all samples and then run through the detection process (31).

The metabolomics analysis was carried out using GC (7890B; Agilent Technologies, Palo Alto, CA, USA) coupled to a TOF-MS (Pegasus BT, LECO Corporation, Saint Joseph, MI, USA). GC was performed on a DB-5MS capillary column (30 m ×250 μm i.d., 0.25 μm film thickness, Agilent Technologies, Palo Alto, CA, USA) to separate the derivatives at a constant flow of 1 mL/min helium. One μL of sample was injected by the auto-sampler with a split/splitless ratio of 10:1. The temperatures for injection, transfer line and ion source were 280°, 320°, and 230°C, respectively. The initial oven temperature was maintained at 50°C for 0.5 min, increased with 15°C/min to 320°C, and then maintained at 320°C for 9 min. The transfer line between GC and MS was maintained at 250°C. MS was operated using a full scan method with an m/z range of 40–650 at a rate of 10 spec/s in electron ionization mode at −70 eV, and a solvent delay was set to 3 min. Raw data were annotated based on the Fiehn Library (https://fiehnlab.ucdavis.edu/).

### Lipid Extraction

Lipids were extracted from the samples with chloroform/methanol (2:1, v/v) and ground using a high-flux tissue grinder (Xinzhi Biotechnology Co., Ltd, Ningbo, China). The homogenate was placed on ice for 2 h, centrifuged at 12,000 × g for 10 min at 4°C, and then the resultant supernatant was blown dry using a nitrogen blowing instrument (BLDCY-12Y, Biao long Instrument Co., Ltd, Shanghai, China). The lipids were stored at −80°C for further analysis.

### Fatty Acid Detection

Fatty acid profiling was performed according to a previously published protocol (32). Briefly, lipids were converted into FAMEs using a mixture of 0.50 mol/L potassium hydroxide, methanol, and boron trifluoride etherate in a water bath at 65°C for 60 min. FAME profiles were determined by GC (7890B, Agilent Technologies, Palo Alto, CA, USA) equipped with a CP7487 capillary column (60 m × 250 μm i.d., 0.20 μm film thickness, Agilent Technologies, Palo Alto, CA, USA) and a hydrogen flame ionization detector to determine the fatty acid profiles of the meat samples and FAME mix standards. GC was set to the initial column oven temperature was kept at 140°C for 5 min, increased with 4°C/min to 220°C, and maintained at 220°C for 10 min. Fatty acids were identified by comparing FAME mix standards.

### Lipids Detection

The lipids were dissolved by 200 μL isopropanol and filtered through a 0.22 μm membrane to obtain the prepared samples for liquid chromatography-mass spectrometry (LC-MS). From each sample, 20 μL was retained as QC samples used to monitor deviations of the analytical results.

LC-MS was performed as previously described (30). Briefly, liquid chromatography (UltiMate 3000, Thermo Fisher Scientific, CA, USA) was coupled to a mass spectrometer (Q Exactive Focus, Thermo Fisher Scientific, CA, USA) and equipped with an acquity uplc beh c18 column (100 × 2.1 mm, 1.7 μm, Waters Technologies Limited, Shanghai, China) maintained at 50 °C. The temperature of the autosampler was set to 8°C. Gradient elution of analytes was carried out with a 60:40 mixture of acetonitrile and water (0.1% formic acid + 10 mM ammonium formate; solvent C) and a 90:10 mixture of isopropanol and acetonitrile (0.1% formic acid + 10 mM ammonium formate; solvent D) at a flow rate of 0.25 mL/min. Injection of 2 μL of each sample was done after equilibration. An increasing linear gradient of solvent C (v/v) was used as follows: 0–5 min, 70–57% C; 5–5.1 min, 57–50% C; 5.1–14 min, 50–30% C; 14–14.1 min, 30% C; 14.1–21 min, 30–−1% C; 21–24 min, 1% C; 24–24.1 min, 1–70% C; 24.1–28 min, 70% C.

MS was equipped with an electrospray ion source (ESI) and the spray voltage was 3.5 and −2.5 kV in positive and negative modes, respectively. Sheath gas and auxiliary gas were set at 30 and 10 arbitrary units, respectively. The capillary temperature was 325°C. The Orbitrap analyzer scanned over a mass range of m/z 150–2,000 for full scan at a mass resolution of 35,000. Data-dependent acquisition was performed for MS/MS acquisition. The normalized collision energy was 30 eV. Dynamic exclusion was implemented to remove noise in MS/MS spectra.

### Data Analysis

Data were analyzed by unpaired *t*-test using the Prism 7.0 software (GraphPad) and represented as means ± standard error of the mean (SEM, *n* = 6). Significant differences were defined as *p* < 0.05. Multivariate statistical analyses including principal component analysis (PCA) and orthogonal projections to latent structures discriminant analysis (OPLS-DA) were used to assess metabonomics and lipidomics data, respectively. A variable importance in projection (VIP) score of >1 and *p* < 0.05 were used to identify differential metabolites and lipids. Receiver operating characteristic curves (ROC) were used to screen for potential biomarkers for the discrimination of samples.

## Results

### Metabolic Profiles in Cooked and Raw Donkey Meat

**Figure 1:**
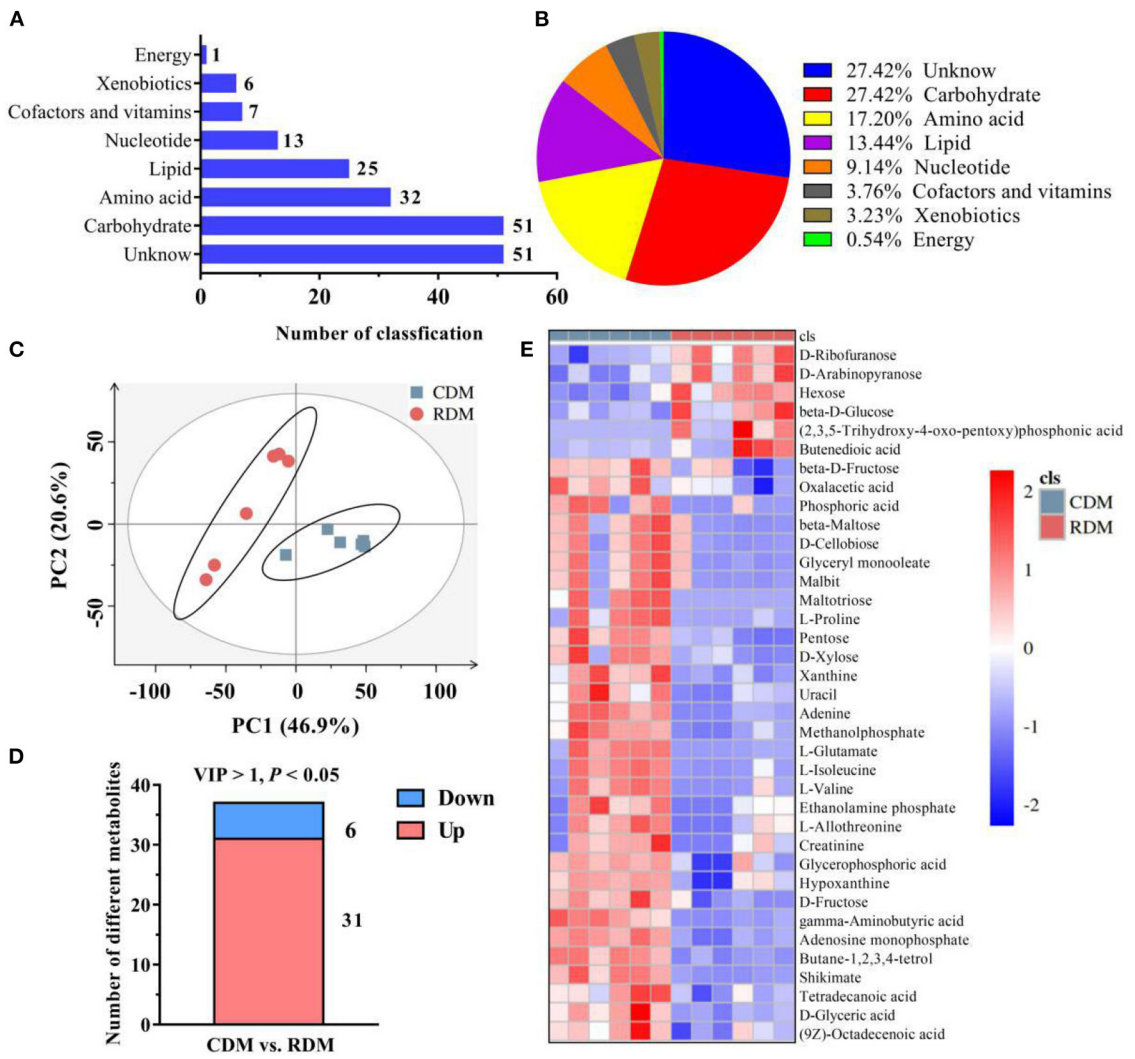
Overall metabolic profiles and different metabolites in cooked and raw donkey muscles. **(A,B)** The number **(A)** and percentage **(B)** of metabolites in muscles. **(C)** Principal component analysis (PCA) based on metabolic data from cooked and raw muscles. **(D)** The number of significantly different metabolites in CDM vs. RDM. **(E)** Heatmap analysis of different metabolites.

Supplementary Figure 1 shows representative base peak diagrams of GC-MS. A total of 186 metabolites belonging to 8 subclasses were identified in CDM and RDM (Figure 1A). These metabolites included amino acids (17.20%), carbohydrates (27.42%), nucleotides (9.14%), cofactors and vitamins (3.76%), lipids (13.44%), energy metabolites (0.54%), xenobiotics (3.23%), and unclassified metabolites (27.42%; Figure 1B). The PCA based on metabolite data showed a clear separation between CDM and RDM (Figure 1C). In total, 37 significantly different metabolites were identified between the groups (Figure 1D, Supplementary Table 1; VIP > 1; *p* < 0.05). Among the top metabolites with largest fold change in CDM vs. RDM were maltotriose (346.31), L-glutamate (35.26), and L-proline (20.26; Supplementary Table 1). Agglomerative hierarchical clustering was subsequently applied to analyze the differential metabolites identified above. As shown in Figures 1D,E, 31 metabolites were upregulated in CDM, including 8 amino acids, 7 carbohydrates, 5 lipids, 5 nucleotides, 5 unclassified metabolites, and 1 xenobiotics, while 6 metabolites were downregulated in CDM, comprising 4 carbohydrates and 2 unknown metabolites.

### Potential Metabolic Marker Compounds in Cooked and Raw Donkey Meat

**Figure 2:**
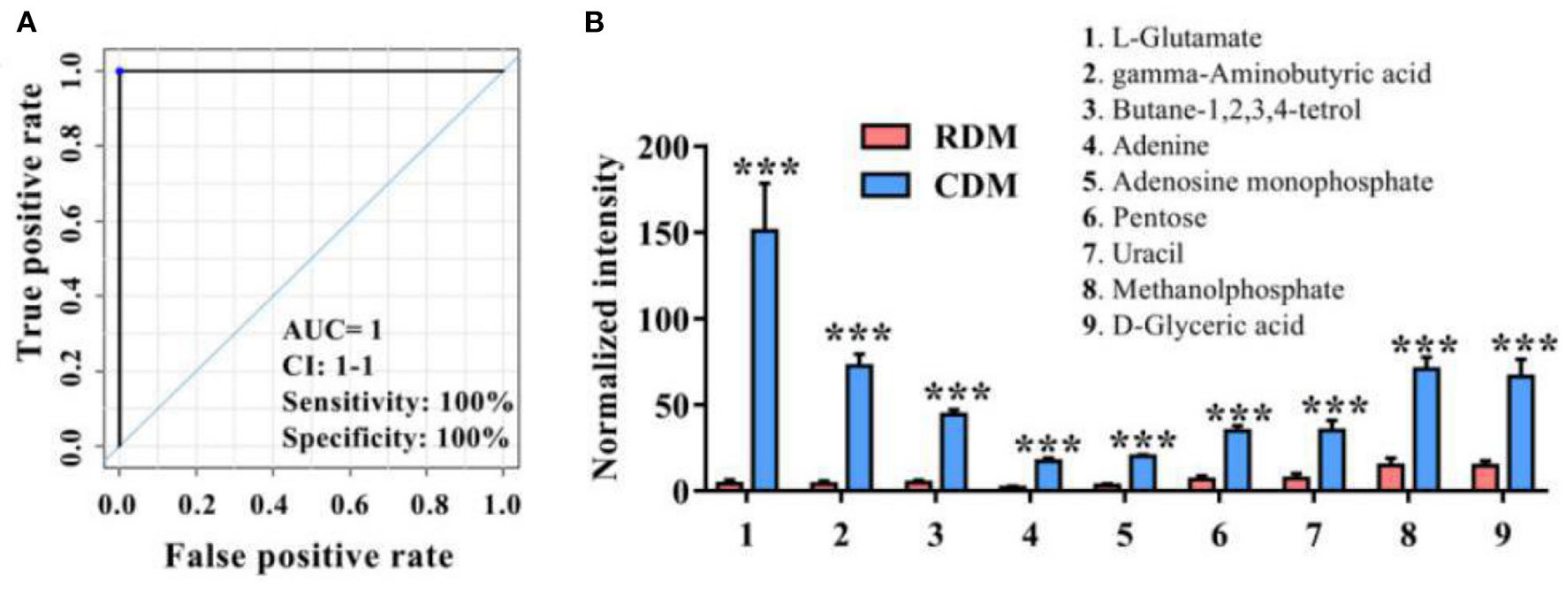
Potential metabolic marker compounds in cooked and raw donkey muscles. **(A)** Receiver operating characteristic curve of **(B)**. **(B)** Normalized intensity for potential metabolic marker compounds in RDM and CDM. Data are presented as means ± SEM (*n* = 6), ***p < 0.001. AUC is the area under the ROC curve; CI 1–1 is the lower and upper limit of the AUC confidence interval.

Figure 2A and Supplementary Table 2 show ROC curves and parameters for the top 9 discriminating metabolites, with an area under the ROC curve (AUC) of 1, specificity of 100%, and sensitivity of 100% for L-glutamate, gamma-aminobutyric acid, butane-1,2,3,4-tetrol, adenine, adenosine monophosphate, pentose, uracil, methanolphosphate and d-glyceric acid. The normalized intensities of these metabolites were significantly higher in CDM than in RDM (*p* < 0.001; Figure 2B).

### Lipid Profiles in Cooked and Raw Donkey Meat

**Figure 3:**
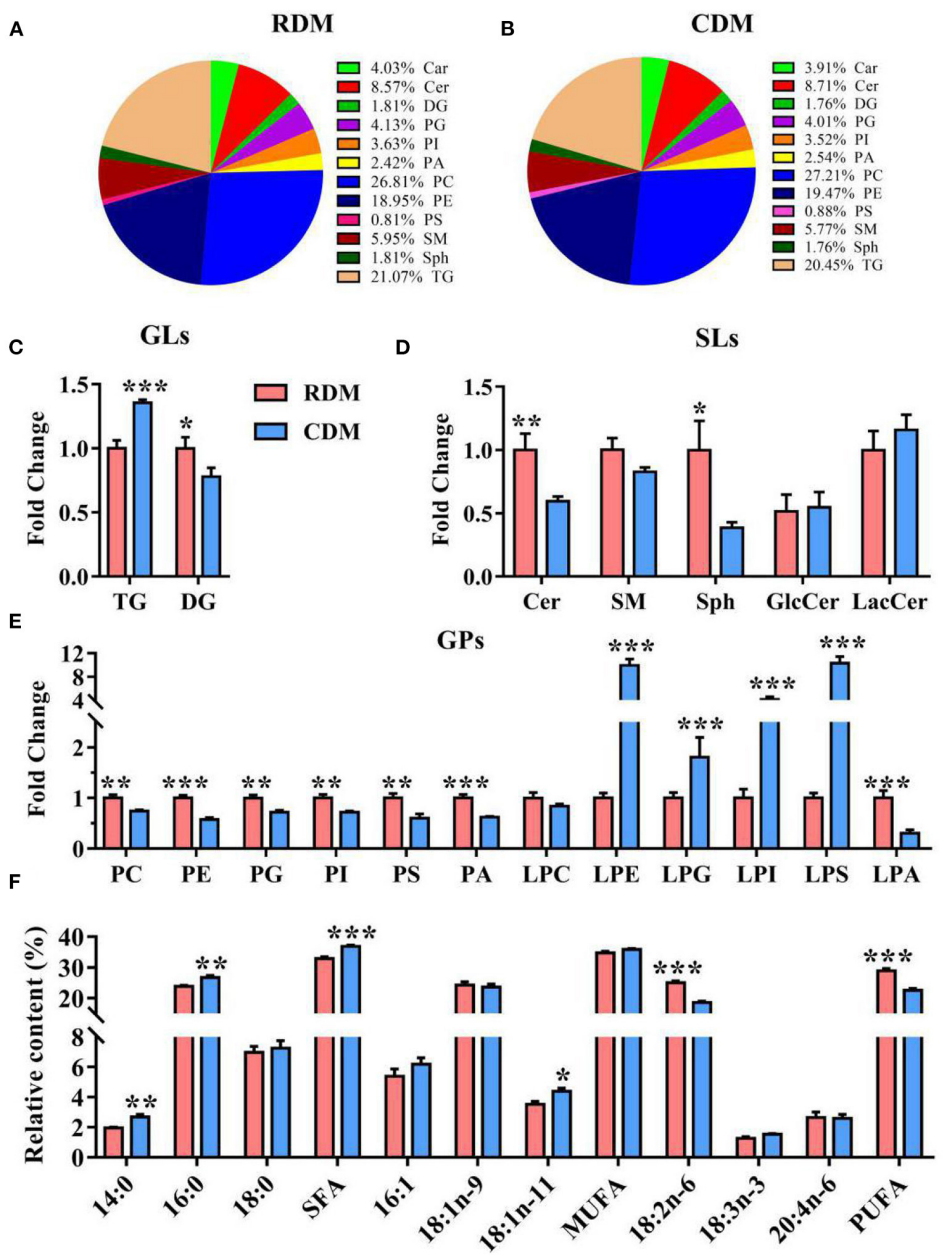
Overall lipid profiles in cooked and raw donkey muscles. **(A,B)** Percentage of lipid subclasses in RDM **(A)** and CDM **(B)**. **(C–E)** Relative content (% of total lipids) of GLs **(C)**, SPs **(D)** and GPs **(E)** in cooked and raw donkey muscles. **(F)** Fatty acid profiles in cooked and raw donkey muscles. Data are presented as means ± SEM (*n* = 6), *p < 0.05, **p < 0.01, ***p < 0.001. GLs, Glycerolipids; SLs, Sphingolipids; GPs, Glycerophospholipids.

Qualitative lipid analysis achieved excellent separation between CDM and RDM in positive and negative mode (Supplementary Figures 2A,B). A total of 992 lipids were identified in RDM and 1,022 lipids were identified in CDM. These lipids belonged to 12 subclasses: carnitines (Car), ceramides (Cer), diglycerides (DG), phosphatidylglycerols (PG), phosphatidylinositols (PI), phosphatidic acid (PA), phosphatidylcholines (PC), phosphatidylethanolamines (PE), phosphatidylserines (PS), sphingomyelins (SM), sphingosines (Sph), and TG (Figures 3A,B). The relative content of TG in CDM was significantly higher than in RDM (*p* < 0.001), whereas the opposite was true for DG (*p* < 0.05; Figure 3C). The relative contents of Cer and Sph in CDM were significantly lower than in RDM (*p* < 0.01; *p* < 0.05; Figure 3D). The relative contents of PC, PE, PG, PI, PS, PA, and lysophosphatidic acid (LPA) were significantly lower in CDM than in RDM (*p* < 0.01), whereas the opposite was true for lysophosphatidylethanolamine (LPE), lysophosphatidylglycerol (LPG), lysophosphatidylinositol (LPI), and lysophosphatidylserine (LPS; *p* < 0.01; Figure 3E). The relative contents of the fatty acids 14:0, 16:0, SFA, and 18:1n-11 were significantly higher in CDM than in RDM (*p* < 0.05), whereas the opposite was true for 18:2n-6 and PUFA (*p* < 0.001; Figure 3F).

### Differential Lipid Molecules in Cooked and Raw Donkey Meat

**Figure 4:**
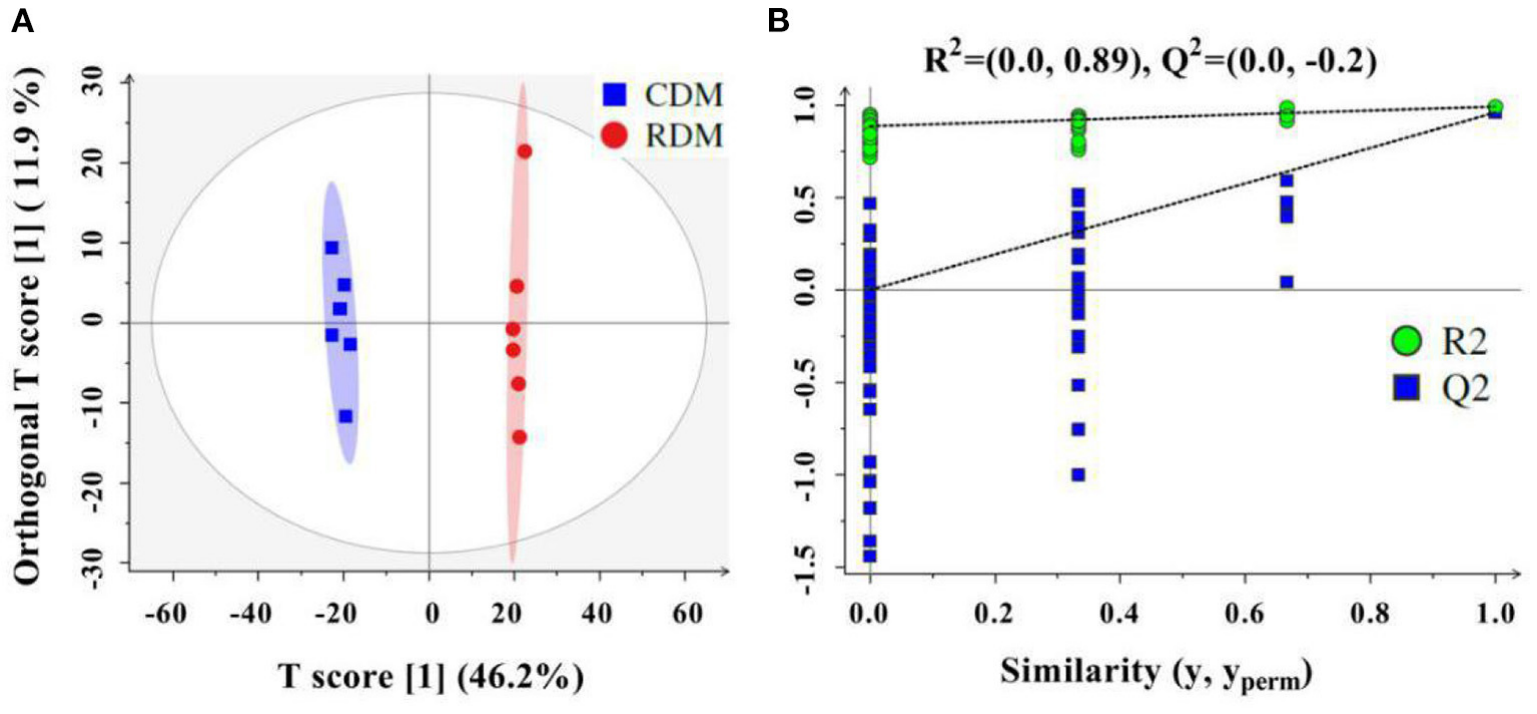
Orthogonal projections to latent structures discriminant analysis (OPLS-DA) in cooked and raw donkey muscles. **(A)** OPLS-DA score plots based on lipidomic data from cooked and raw donkey muscles (*R*^2^*X* = 0.581, *R*^2^*Y* = 0.996, *Q*^2^ = 0.95) and **(B)** corresponding OPLS-DA validation plots (*R*^2^ = (0.0, 0.89), *Q*^2^ = (0.0, −0.20).

**Figure 5:**
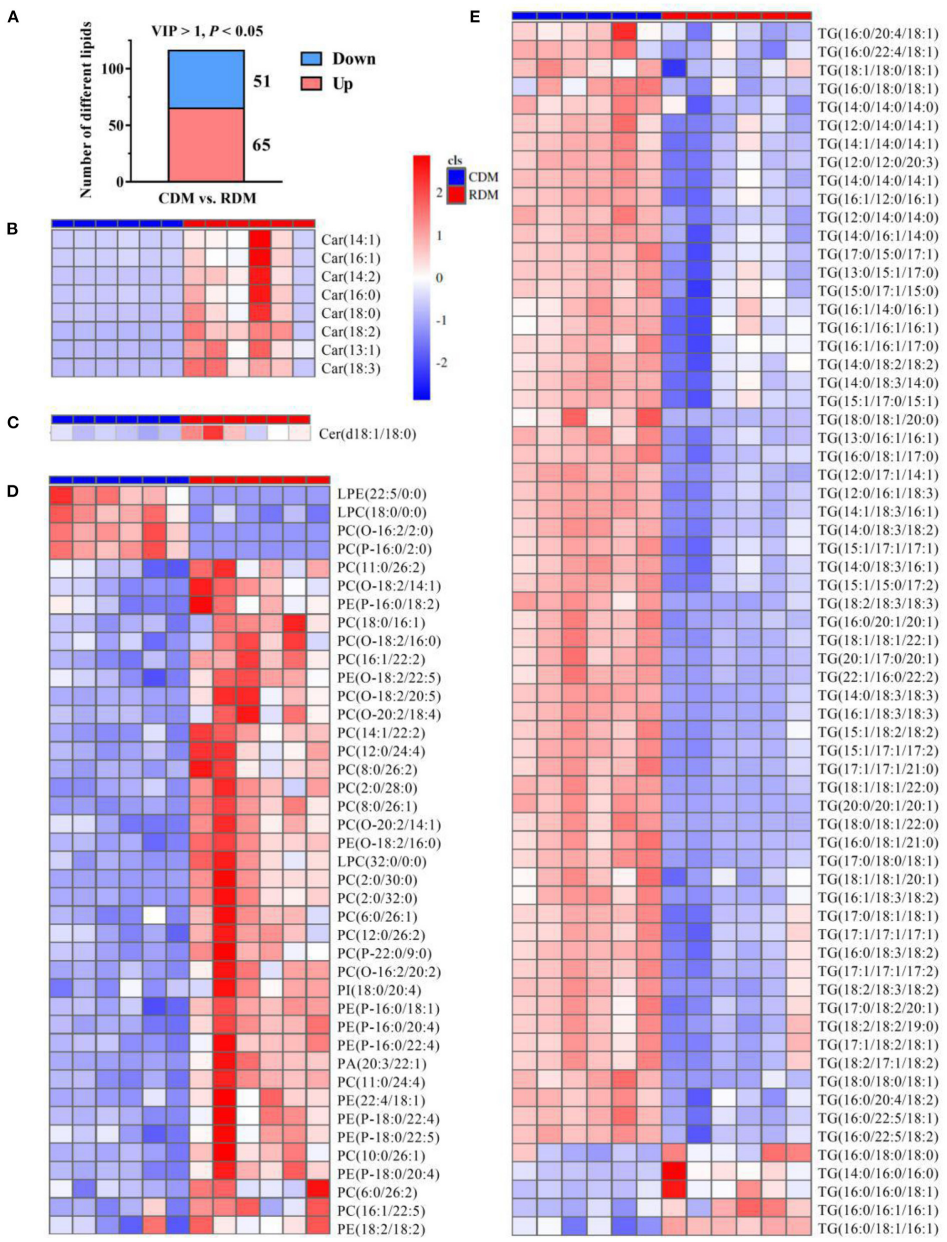
Differential lipids in cooked and raw donkey muscles. **(A)** The number of significantly differential lipids in CDM vs. RDM. **(B–E)** Heatmap analysis of carnitines [Cars; **(B)**], ceramides [Cers; **(C)**], glycerophospholipids [GPs; **(D)**], and triglycerides [TGs; **(E)**].

The OPLS-DA showed that there were clear differences between raw and cooked donkey meat (R^2^X = 0.581, R^2^Y = 0.996, Q^2^ = 0.95; Figure 4A). The corresponding OPLS-DA validation plots showed satisfactory *R*^2^ (0.0, 0.89) and *Q*^2^ (0.0, −0.20) scores (Figure 4B). In total, 116 lipid molecules were identified as significantly different between CDM and RDM by setting VIP > 1 and *p* < 0.05 (Supplementary Table 3). Among these, 51 lipids were downregulated in CDM, including 8 Cars, 1 Cer, 1 LPC, 1 PA, 23 PCs, 11 PEs, 1 PI, and 5 TGs, while 65 lipids were upregulated in CDM, including 1 LPE, 1 LPC, 2 PCs, and 61 TGs (Figures 5A–E).

### Potential Lipid Markers in Cooked and Raw Donkey Meat

**Figure 6:**
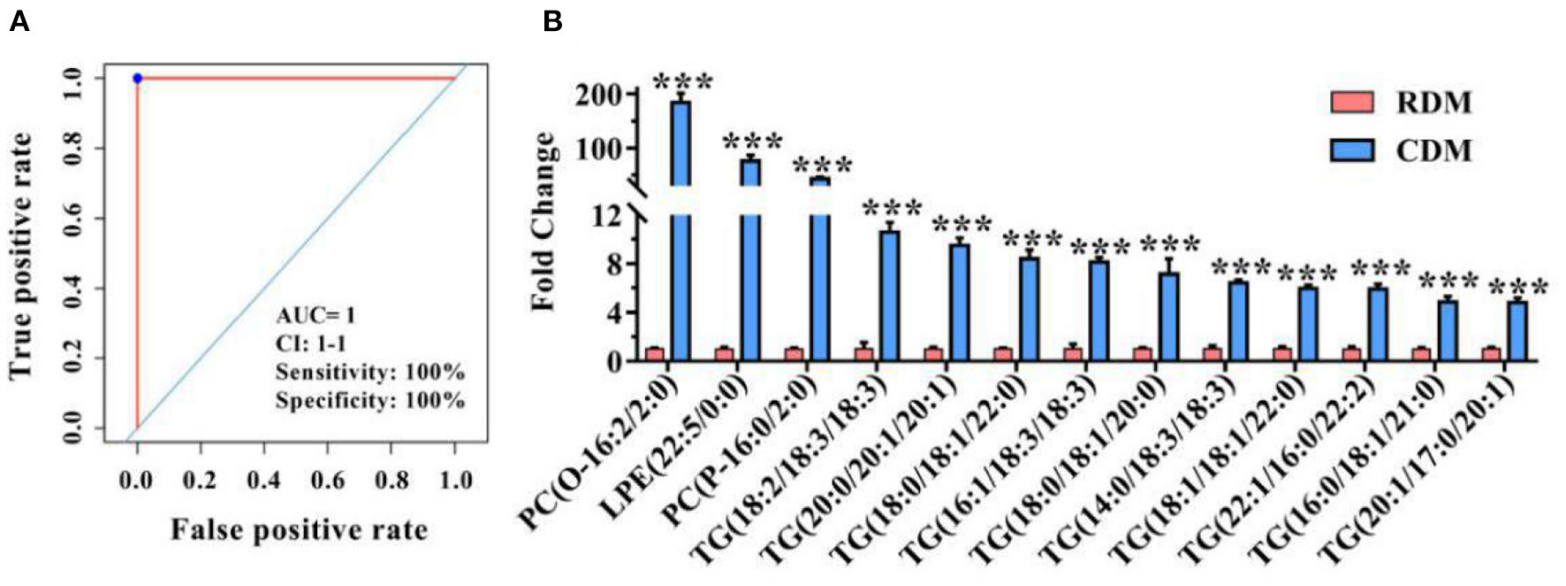
Potential lipid markers in cooked and raw donkey muscles. **(A)** Receiver operating characteristic curve of **(B)**. **(B)** Fold changes of potential lipid markers in CDM vs. RDM. Data are presented as means ± SEM (*n* = 6), ***p < 0.001. AUC is the area under the ROC curve; CI 1–1 is the lower and upper limit of the AUC confidence interval.

Figure 6A and Supplementary Table 4 show ROC curves and parameters for the top 13 discriminating lipids, with an area under the ROC curve (AUC) of 1, specificity of 100%, and sensitivity of 100% for 2 PCs, 1 LPE, and 10 TGs, for which the normalized intensity was significantly higher in CDM than in RDM (*p* < 0.001; Figure 6B).

## Discussion

In the present study, a total of 186 metabolites belonging to 8 subclasses including amino acid, carbohydrates, nucleotides, cofactors and vitamins, lipids, energy metabolites, and xenobiotics were identified in raw and cooked donkey meat. These results are in line with a previous study detecting 188 metabolites in pork using MS-based metabolomics (27 metabolites), but significantly higher than the number of metabolites previously found in chicken (25 metabolites), cow (37 metabolites), goat (37 metabolites), and donkey (37 metabolites) using nuclear magnetic resonance (NMR) assessment (33, 34). The PCA results showed a clear separation between CDM and RDM, and 37 significantly different metabolites were identified, including 8 amino acids, 11 carbohydrates, 5 lipids, 5 nucleotides, and 1 xenobiotics. These findings further indicate that heat treatment significantly affects the nutritional composition of meat, especially the composition of amino acids, carbohydrates, and lipids. This is also supported by the fact that high cooking temperature induces the Maillard reaction of carbonyl compounds and lipid oxidation (35). Furthermore, this heating-induced change in nutritional composition may explain the altered taste of cooked meat as taste substances in meat products mainly include amino acids, small peptides, sugars, nucleic acid metabolites, vitamins, lipids, and inorganic salts (35). Further analysis showed that 31 of the 37 differential metabolites were upregulated in CDM vs. RDM, and that the top 3 metabolites with highest fold change were maltotriose, L-glutamate, and L-proline. Meat contains a certain amount of sugars, among maltotriose is stable at high temperature and is not prone to Maillard reaction due to it has heat resistance. The protein in meat is degraded into free amino acids during heat cooking, some amino acids generate volatile compounds through Maillard reaction and Strecker degradation, and the other part is retained to form the taste of meat, especially for glutamate (36). While proline is formed during Maillard reaction, which produces the roasty and sweet flavor in cooked meat (37). Previous studies have shown that maltotriose and L-proline are responsible for sweet taste, while glutamate contributes to umami taste (16). Therefore, these substances might predominantly contribute to the unique umami and sweet taste of cooked donkey meat. In addition, 9 metabolites including L-glutamate, gamma-aminobutyric acid, butane-1,2,3,4-tetrol, adenine, adenosine monophosphate, pentose, uracil, methanolphosphate and d-glyceric acid were analyzed by ROC curves and found to accurately predict the CDM state, indicating that these metabolites may serve as potential biomarkers for the discrimination of raw and cooked donkey meat.

In the present study, LC-MS based lipidomics profiling was performed to characterize the lipid composition of donkey meat, and a total of 992 and 1,022 lipids belonging to 12 subclasses were identified in RDM and CDM, respectively, mainly comprising GLs, GPs, and SLs. These findings are in accordance with the results from previous report both with regards to the amount and types of lipids identified (16). Lipids are considered the key aroma retainers due to the lipophilic nature of most aroma compounds (18). High lipid contents could increase the partition coefficients of aroma compounds, promoting the aroma retention (18), and TG might predominantly contribute to the aroma retention owe to the high species and concentrations (22). In the present study, the content of TG in CDM was found to be significantly higher than in RDM, suggesting that TG may play an important role in contributing to the aroma retention of donkey meat. Cooking increased the content of TG in meat, which may be due to the increase of the relative content of SFA, while the degradation of PUFA and consequence of moisture loss (38). A series of chemical reactions such as lipid degradation and oxidation occur in lipids during heating, and this is especially the case for GPs and SLs owing to their being rich in PUFAs (22). Meat GPs are more important substances for flavor formation than proteins and carbohydrates (18). In line with this, the current study found that the contents of GP and SL were significantly lower in CDM compared to RDM, especially the contents of PC and PE. Furthermore, an opposite trend was found for LPE, LPG, LPI, and LPS, indicating that the hydrolysis of GPs could generate LPE, LPG, LPI, and LPS (39). The production of flavor compounds is linked to the PUFA contents. The relative contents of 18:2n-6 and PUFA in CDM were significantly lower than in RDM (40), which further suggests that the PUFAs in donkey meat may be an important precursor of flavor compounds.

OPLS-DA-based supervised chemometric assessment of lipid profiles revealed a clear separation between RDM and CDM, and no overfitting of the OPLS-DA model occured. This is in accordance with a recent study reporting that the OPLS-DA model can discriminate between different samples (40). In the present study, a total of 116 significantly different lipid molecules were identified, among which 37 GPs (1 LPC, 1 PA, 23 PCs, and 11 PEs) were downregulated and 61 TGs were upregulated in CDM relative to RDM. Interestingly, the downregulated GPs were mainly composed of PUFAs, such as fatty acids 18:2, 20:4, 20:5, 22:2, 22:5, and 24:4. The degradation of fatty acid 20:4 could produce 1-Octen-3-ol (41). Indeed, PUFAs are easy to oxidation products had dominated flavor compounds in meat products during cooking (42, 43). Previous studies have shown that TGs, including TG (16:0_18:1_18:1) and TG (18:0_18:0_18:1), might be the predominant lipids for binding aroma compounds (22). In the present study, the TGs rich in SFA and MUFA were retained in cooked meat, which might crucially contribute to the aroma retention in CDM.

The lipidomic data were analyzed by ROC curves to screen for potential biomarkers, and the results indicated that PC(O-16:2/2:0), LPE(22:5/0:0), and PC(P-16:0/2:0) could satisfactorily distinguish between RDM and CDM. Previous studies have identified PC as a potential marker for the discrimination of roasted mutton (22), meat from castrated and uncastrated lambs (44), and donkey intramuscular fat and visceral adipose tissue (20). Thus, the current results demonstrate that LC-MS based lipidomics together with ROC analysis is a promising approach for the differentiation of cooked donkey meat.

## Conclusion

In the present work, heat cooking treatment of donkey meat led to an altered metabolite and lipid composition. In particular, the contents of maltotriose, L-glutamate, and L-proline significantly increased in CDM, and these substances are likely responsible for the more intense umami and sweet taste found in CDM compared to RDM. In addition, the abundances of TGs rich in SFAs and MUFA were retained, while the abundances of GPs rich in PUFAs were reduced in CDM, suggesting that TGs and GPs might be the predominant lipids for binding and generating aroma compounds, respectively. In conclusion, this study provides useful information about the dynamic composition of metabolites and lipids in donkey meat which may explain its unique taste and flavor. However, the changes of metabolite, lipid and volatile compounds in donkey meat during heat cooking are not clear and require further studies.

## Data Availability Statement

The original contributions presented in the study are included in the article/Supplementary Material, further inquiries can be directed to the corresponding author.

## Author Contributions

ML: conceptualization, investigation, and writing and editing the original draft. WR: methodology, formal analysis, and writing and editing the original draft. WC: methodology, writing, and reviewing and editing. MZ, LM, YZ, HQ, MS, JL, DZ, YW, TW, and XS: software, investigation, formal analysis, and resources. CW: funding acquisition, supervision, writing, and review and editing. All authors contributed to the article and approved the submitted version.

## Funding

This work was supported by the Open Project of Liaocheng Universtiy Animal Husbandry Discipline (319312101–10), the Scientific Research Fund of Liaocheng University (318052019), the Shandong Province Modern Agricultural Technology System Donkey Industrial Innovation Team (SDAIT-27), the Taishan Leading Industry Talents-Agricultural Science of Shandong Province (LJNY201713), and the Open Project of Shandong Collaborative Innovation Center for Donkey Industry Technology (319330811).

## Conflict of Interest

The authors declare that the research was conducted in the absence of any commercial or financial relationships that could be construed as a potential conflict of interest.

## Publisher's Note

All claims expressed in this article are solely those of the authors and do not necessarily represent those of their affiliated organizations, or those of the publisher, the editors and the reviewers. Any product that may be evaluated in this article, or claim that may be made by its manufacturer, is not guaranteed or endorsed by the publisher.
