# Supplementary material for: Comparing the Profiles of Raw and Cooked Donkey Meat by Metabonomics and Lipidomics Assessment
Source: Front Nutr. 2022 Mar 25;9:851761. doi: 10.3389/fnut.2022.851761 (PMC8990316; doi:10.3389/fnut.2022.851761)
Supplement: Supplementary file 1 [file Data_Sheet_1.doc]

**Supplemental file**


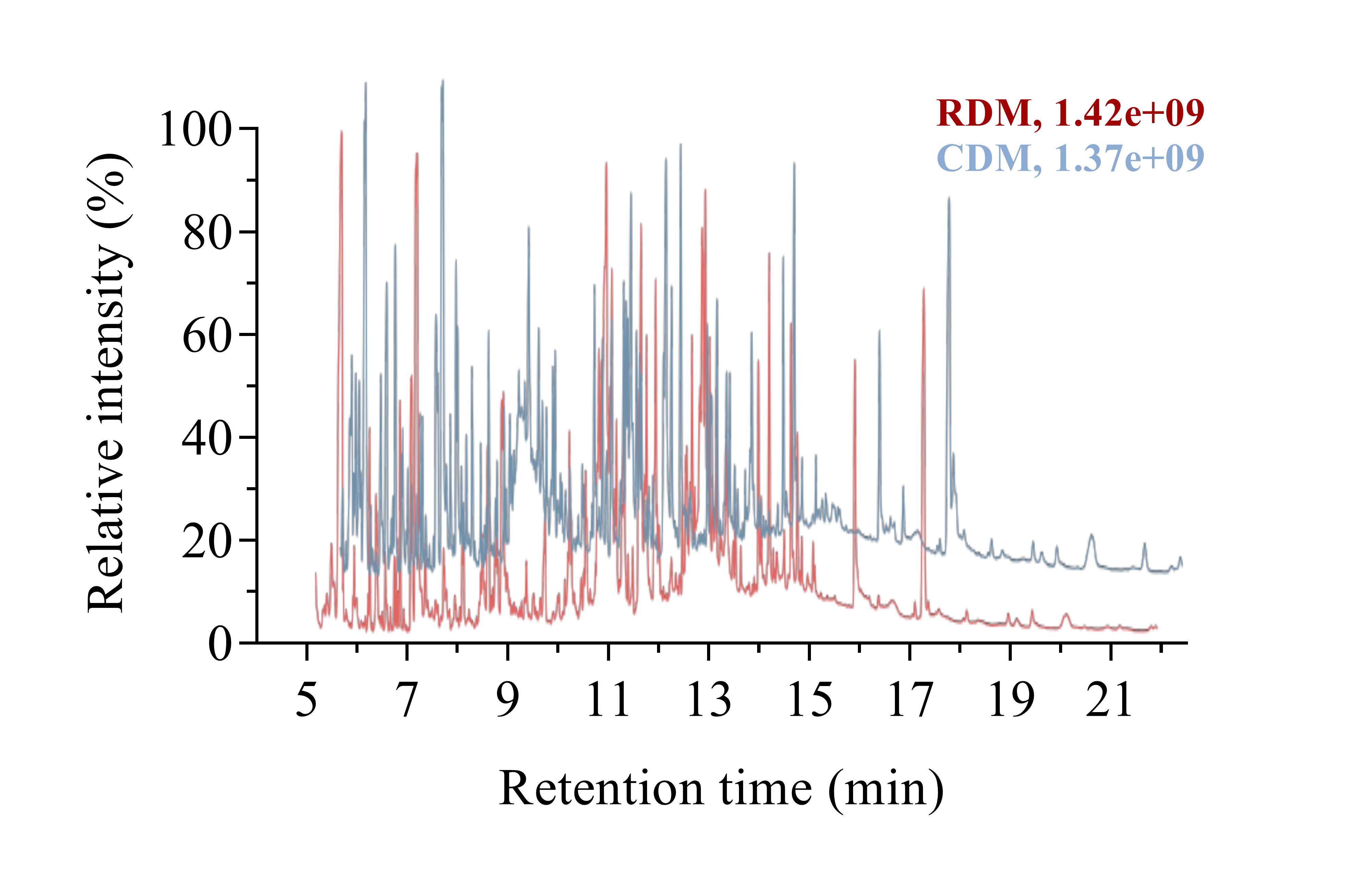
**Fig. S1 Representative base peak diagrams of GC-MS**


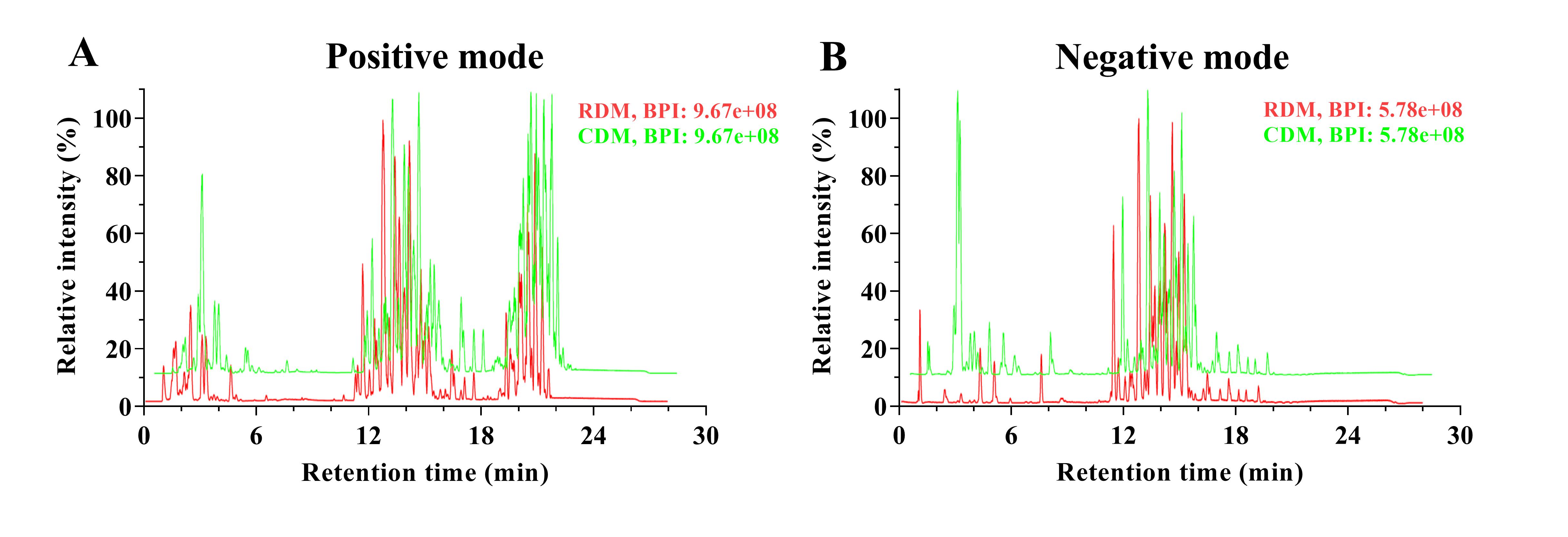
**Fig. S2 Representative base peak diagrams.** (A and B) Representative base peak diagrams of electrospray ionisation in positive (A) and negative (B) modes.

**Table S1 Information on different metabolites in cook and raw of donkey muscles**

| No. | Name | Class | Rt(min) | *mz* | VIP | FC | log2(FC) | p.value | FDR |
| --- | --- | --- | --- | --- | --- | --- | --- | --- | --- |
| 1 | Shikimate | Amino acid | 10.497 | 204.1998 | 2.92 | 2.20 | 1.14 | 0.0000 | 0.0000 |
| 2 | gamma-Aminobutyric acid | Amino acid | 8.950 | 174.1896 | 2.23 | 17.74 | 4.15 | 0.0000 | 0.0001 |
| 3 | L-Glutamate | Amino acid | 9.457 | 246.2694 | 3.11 | 35.26 | 5.14 | 0.0003 | 0.0024 |
| 4 | L-Isoleucine | Amino acid | 7.356 | 158.2371 | 2.78 | 9.80 | 3.29 | 0.0017 | 0.0090 |
| 5 | L-Proline | Amino acid | 7.424 | 142.1883 | 2.43 | 20.26 | 4.34 | 0.0051 | 0.0164 |
| 6 | L-Valine | Amino acid | 6.799 | 144.2009 | 3.13 | 6.19 | 2.63 | 0.0059 | 0.0173 |
| 7 | L-Allothreonine | Amino acid | 7.965 | 218.2388 | 1.27 | 3.07 | 1.62 | 0.0266 | 0.0531 |
| 8 | Creatinine | Amino acid | 9.131 | 115.1380 | 2.19 | 2.98 | 1.57 | 0.0428 | 0.0813 |
| 9 | D-Fructose | Carbohydrate | 10.831 | 103.1002 | 1.75 | 1.41 | 0.49 | 0.0002 | 0.0018 |
| 10 | Hexose | Carbohydrate | 11.335 | 204.2026 | 2.65 | 0.30 | -1.74 | 0.0003 | 0.0024 |
| 11 | D-Ribofuranose | Carbohydrate | 9.752 | 103.0994 | 1.88 | 0.36 | -1.46 | 0.0004 | 0.0028 |
| 12 | D-Glyceric acid | Carbohydrate | 7.574 | 147.1427 | 1.82 | 4.53 | 2.18 | 0.0006 | 0.0037 |
| 13 | D-Arabinopyranose | Carbohydrate | 9.753 | 103.1011 | 1.60 | 0.51 | -0.97 | 0.0014 | 0.0076 |
| 14 | D-Xylose | Carbohydrate | 9.664 | 103.0976 | 1.25 | 5.29 | 2.40 | 0.0019 | 0.0094 |
| 15 | Maltotriose | Carbohydrate | 21.282 | 204.1985 | 2.39 | 346.31 | 8.44 | 0.0026 | 0.0107 |
| 16 | beta-D-Glucose | Carbohydrate | 11.086 | 205.2217 | 1.11 | 0.76 | -0.39 | 0.0058 | 0.0173 |
| 17 | beta-D-Fructose | Carbohydrate | 10.901 | 147.1410 | 1.05 | 1.44 | 0.52 | 0.0098 | 0.0261 |
| 18 | D-Cellobiose | Carbohydrate | 14.684 | 361.3360 | 2.06 | 5.15 | 2.36 | 0.0132 | 0.0323 |
| 19 | Oxalacetic acid | Carbohydrate | 5.579 | 174.1585 | 1.22 | 1.97 | 0.98 | 0.0134 | 0.0324 |
| 20 | Glycerophosphoric acid | Lipid | 10.243 | 299.2467 | 1.74 | 2.48 | 1.31 | 0.0039 | 0.0138 |
| 21 | Tetradecanoic acid | Lipid | 10.798 | 117.0854 | 1.12 | 2.62 | 1.39 | 0.0057 | 0.0173 |
| 22 | (9Z)-Octadecenoic acid | Lipid | 12.587 | 117.0847 | 1.17 | 1.66 | 0.73 | 0.0069 | 0.0194 |
| 23 | Phosphoric acid | Lipid | 7.203 | 299.2318 | 3.11 | 2.25 | 1.17 | 0.0076 | 0.0210 |
| 24 | Ethanolamine phosphate | Lipid | 10.405 | 299.2257 | 1.19 | 3.09 | 1.63 | 0.0298 | 0.0590 |
| 25 | Adenosine monophosphate | Nucleotide | 16.420 | 169.1418 | 1.12 | 6.31 | 2.66 | 0.0000 | 0.0000 |
| 26 | Adenine | Nucleotide | 10.932 | 264.2450 | 1.04 | 8.46 | 3.08 | 0.0000 | 0.0003 |
| 27 | Xanthine | Nucleotide | 11.629 | 147.1282 | 1.54 | 4.29 | 2.10 | 0.0003 | 0.0024 |
| 28 | Uracil | Nucleotide | 7.678 | 241.2249 | 1.31 | 4.89 | 2.29 | 0.0018 | 0.0091 |
| 29 | Hypoxanthine | Nucleotide | 10.587 | 265.2423 | 2.43 | 2.30 | 1.20 | 0.0045 | 0.0150 |
| 30 | Pentose | Unknow | 9.665 | 103.0989 | 1.41 | 5.30 | 2.41 | 0.0000 | 0.0005 |
| 31 | Methanolphosphate | Unknow | 6.510 | 241.2092 | 1.97 | 4.75 | 2.25 | 0.0000 | 0.0006 |
| 32 | beta-Maltose | Unknow | 14.677 | 361.3441 | 2.16 | 5.16 | 2.37 | 0.0068 | 0.0194 |
| 33 | Glyceryl monooleate | Unknow | 14.804 | 147.1324 | 1.18 | 4.52 | 2.18 | 0.0116 | 0.0298 |
| 34 | Malbit | Unknow | 14.796 | 204.1949 | 1.45 | 5.14 | 2.36 | 0.0117 | 0.0298 |
| 35 | (2,3,5-Trihydroxy-4-oxo-pentoxy)phosphonic acid | Unknow | 12.044 | 357.2884 | 1.08 | 0.01 | -6.29 | 0.0178 | 0.0399 |
| 36 | Butenedioic acid | Unknow | 7.744 | 245.2108 | 1.79 | 0.15 | -2.78 | 0.0385 | 0.0738 |
| 37 | Butane-1,2,3,4-tetrol | Xenobiotics | 8.732 | 147.1363 | 1.71 | 9.20 | 3.20 | 0.0000 | 0.0000 |

Rt, retention time; VIP, variable importance in projection; FC, Fold change; FDR, false discovery rate.

**Table S2 Receiver operating characteristic parameter of different metabolites in cook and raw of donkey muscles**

| No. | Metabolites name | AUC | Ci1 | Ci2 | Specificity  (%) | Sensitivity  (%) | Threshold |
| --- | --- | --- | --- | --- | --- | --- | --- |
| 1 | L-Glutamate | 1 | 1 | 1 | 1 | 1 | 14.98 |
| 2 | gamma-Aminobutyric acid | 1 | 1 | 1 | 1 | 1 | 30.01 |
| 3 | Butane-1,2,3,4-tetrol | 1 | 1 | 1 | 1 | 1 | 21.65 |
| 4 | Adenine | 1 | 1 | 1 | 1 | 1 | 6.74 |
| 5 | Adenosine monophosphate | 1 | 1 | 1 | 1 | 1 | 11.58 |
| 6 | Pentose | 1 | 1 | 1 | 1 | 1 | 20.04 |
| 7 | Uracil | 1 | 1 | 1 | 1 | 1 | 17.76 |
| 8 | Methanolphosphate | 1 | 1 | 1 | 1 | 1 | 39.08 |
| 9 | D-Glyceric acid | 1 | 1 | 1 | 1 | 1 | 34.31 |

Area under the receiver operating characteristic (ROC) curve (AUC) was the area under ROC curve, Ci1 is the lower limit of AUC confidence interval; Ci2 is the upper limit of AUC confidence interval.

**Table S3 Information on different lipids in cook and raw of donkey muscles**

| No. | Name | Class | MainIon | *mz* | Rt(s) | VIP | FC | log2(FC) | p.value | FDR |
| --- | --- | --- | --- | --- | --- | --- | --- | --- | --- | --- |
| 1 | Car(13:1) | Car | Car+H | 356.2795 | 88.2461 | 1.20 | 0.0358 | -4.81 | 0.0009 | 0.0033 |
| 2 | Car(14:1) | Car | Car+H | 370.2953 | 100.0852 | 1.57 | 0.0697 | -3.84 | 0.0202 | 0.0363 |
| 3 | Car(14:2) | Car | Car+H | 368.2796 | 86.3173 | 1.19 | 0.0205 | -5.61 | 0.0134 | 0.0263 |
| 4 | Car(16:0) | Car | Car+H | 400.3418 | 183.8325 | 4.05 | 0.0793 | -3.66 | 0.0232 | 0.0411 |
| 5 | Car(16:1) | Car | Car+H | 398.3264 | 132.6490 | 2.35 | 0.0840 | -3.57 | 0.0202 | 0.0363 |
| 6 | Car(18:0) | Car | Car+H | 428.3735 | 277.6700 | 2.84 | 0.0804 | -3.64 | 0.0119 | 0.0237 |
| 7 | Car(18:2) | Car | Car+H | 424.3421 | 177.5390 | 1.46 | 0.0059 | -7.40 | 0.0006 | 0.0025 |
| 8 | Car(18:3) | Car | Car+H | 422.3263 | 131.1245 | 1.36 | 0.0030 | -8.37 | 0.0015 | 0.0047 |
| 9 | Cer(d18:1/18:0) | Cer | Cer+H | 566.5508 | 919.7990 | 1.63 | 0.4625 | -1.11 | 0.0113 | 0.0229 |
| 10 | LPC(18:0/0:0) | LPC | LPC+HCOO | 568.3620 | 293.5660 | 1.14 | 2.3205 | 1.21 | 0.0000 | 0.0003 |
| 11 | LPC(32:0/0:0) | LPC | LPC+H | 720.5903 | 881.6150 | 1.71 | 0.4670 | -1.10 | 0.0010 | 0.0034 |
| 12 | LPE(22:5/0:0) | LPE | LPE-H | 526.2934 | 160.6760 | 2.21 | 76.1890 | 6.25 | 0.0001 | 0.0004 |
| 13 | PA(20:3/22:1) | PA | PA-H | 779.5639 | 829.9430 | 1.36 | 0.4288 | -1.22 | 0.0002 | 0.0012 |
| 14 | PC(10:0/26:1) | PC | PC+H | 788.6160 | 914.5380 | 3.18 | 0.6866 | -0.54 | 0.0012 | 0.0039 |
| 15 | PC(11:0/24:4) | PC | PC+H | 768.5537 | 858.0070 | 3.94 | 0.3697 | -1.44 | 0.0000 | 0.0004 |
| 16 | PC(11:0/26:2) | PC | PC+H | 800.6158 | 890.9210 | 1.32 | 0.6725 | -0.57 | 0.0061 | 0.0138 |
| 17 | PC(12:0/24:4) | PC | PC+H | 782.5692 | 700.7455 | 4.57 | 0.5796 | -0.79 | 0.0016 | 0.0048 |
| 18 | PC(12:0/26:2) | PC | PC+H | 814.6316 | 927.7795 | 1.67 | 0.5692 | -0.81 | 0.0016 | 0.0049 |
| 19 | PC(14:1/22:2) | PC | PC+H | 784.5846 | 772.5020 | 3.45 | 0.5715 | -0.81 | 0.0001 | 0.0009 |
| 20 | PC(16:1/22:2) | PC | PC+H | 812.6161 | 845.8705 | 1.19 | 0.5541 | -0.85 | 0.0000 | 0.0003 |
| 21 | PC(16:1/22:5) | PC | PC+H | 806.5691 | 683.3230 | 1.22 | 0.5186 | -0.95 | 0.0136 | 0.0265 |
| 22 | PC(18:0/16:1) | PC | PC+HCOO | 804.5762 | 834.1760 | 2.64 | 0.7468 | -0.42 | 0.0027 | 0.0071 |
| 23 | PC(2:0/28:0) | PC | PC+H | 706.5370 | 741.9495 | 1.31 | 0.5299 | -0.92 | 0.0005 | 0.0020 |
| 24 | PC(2:0/30:0) | PC | PC+H | 734.5693 | 829.7480 | 3.57 | 0.3587 | -1.48 | 0.0002 | 0.0012 |
| 25 | PC(2:0/32:0) | PC | PC+H | 762.6005 | 911.5970 | 2.18 | 0.4511 | -1.15 | 0.0003 | 0.0014 |
| 26 | PC(6:0/26:1) | PC | PC+H | 732.5537 | 750.0180 | 2.41 | 0.5983 | -0.74 | 0.0020 | 0.0057 |
| 27 | PC(6:0/26:2) | PC | PC+H | 730.5382 | 676.8850 | 1.48 | 0.6989 | -0.52 | 0.0235 | 0.0415 |
| 28 | PC(8:0/26:1) | PC | PC+H | 760.5848 | 834.1460 | 5.66 | 0.6065 | -0.72 | 0.0000 | 0.0001 |
| 29 | PC(8:0/26:2) | PC | PC+H | 758.5692 | 766.2045 | 6.88 | 0.6638 | -0.59 | 0.0024 | 0.0065 |
| 30 | PC(O-16:2/2:0) | PC | PC(O)+HCOO | 564.3302 | 152.8790 | 8.11 | 184.3400 | 7.53 | 0.0000 | 0.0000 |
| 31 | PC(O-16:2/20:2) | PC | PC(O)+HCOO | 812.5818 | 824.5180 | 1.00 | 0.5386 | -0.89 | 0.0010 | 0.0033 |
| 32 | PC(O-18:2/14:1) | PC | PC(O)+H | 714.5437 | 715.3060 | 1.01 | 0.3264 | -1.62 | 0.0021 | 0.0060 |
| 33 | PC(O-18:2/16:0) | PC | PC(O)+HCOO | 788.5815 | 871.7450 | 1.92 | 0.5096 | -0.97 | 0.0094 | 0.0197 |
| 34 | PC(O-18:2/20:5) | PC | PC(O)+H | 790.5743 | 720.8090 | 1.83 | 0.2050 | -2.29 | 0.0017 | 0.0050 |
| 35 | PC(O-20:2/14:1) | PC | PC(O)+HCOO | 786.5657 | 803.9435 | 4.46 | 0.5942 | -0.75 | 0.0005 | 0.0020 |
| 36 | PC(O-20:2/18:4) | PC | PC(O)+HCOO | 836.5815 | 789.1835 | 1.45 | 0.4641 | -1.11 | 0.0058 | 0.0132 |
| 37 | PC(P-16:0/2:0) | PC | PC(P)+HCOO | 566.3462 | 206.2840 | 3.07 | 43.2130 | 5.43 | 0.0000 | 0.0000 |
| 38 | PC(P-22:0/9:0) | PC | PC(P)+H | 704.5587 | 907.0085 | 1.05 | 0.3095 | -1.69 | 0.0014 | 0.0045 |
| 39 | PE(18:2/18:2) | PE | PE-H | 738.5080 | 722.8435 | 1.06 | 0.6457 | -0.63 | 0.0306 | 0.0509 |
| 40 | PE(22:4/18:1) | PE | PE-H | 792.5525 | 887.1570 | 1.28 | 0.2286 | -2.13 | 0.0022 | 0.0062 |
| 41 | PE(O-18:2/16:0) | PE | PE(O)+H | 702.5433 | 899.4945 | 1.71 | 0.4434 | -1.17 | 0.0000 | 0.0003 |
| 42 | PE(O-18:2/22:5) | PE | PE(O)-H | 774.5448 | 816.9030 | 1.18 | 0.5103 | -0.97 | 0.0007 | 0.0027 |
| 43 | PE(P-16:0/18:1) | PE | PE(P)-H | 700.5287 | 899.0075 | 1.95 | 0.5565 | -0.85 | 0.0000 | 0.0003 |
| 44 | PE(P-16:0/18:2) | PE | PE(P)-H | 698.5129 | 831.2740 | 2.44 | 0.6368 | -0.65 | 0.0101 | 0.0210 |
| 45 | PE(P-16:0/20:4) | PE | PE(P)-H | 722.5130 | 812.4975 | 3.59 | 0.4748 | -1.07 | 0.0000 | 0.0002 |
| 46 | PE(P-16:0/22:4) | PE | PE(P)-H | 750.5444 | 871.1240 | 1.67 | 0.3561 | -1.49 | 0.0008 | 0.0030 |
| 47 | PE(P-18:0/20:4) | PE | PE(P)-H | 750.5445 | 894.8265 | 4.84 | 0.4863 | -1.04 | 0.0004 | 0.0019 |
| 48 | PE(P-18:0/22:4) | PE | PE(P)-H | 778.5761 | 947.8280 | 1.43 | 0.4194 | -1.25 | 0.0024 | 0.0065 |
| 49 | PE(P-18:0/22:5) | PE | PE(P)-H | 776.5601 | 893.9040 | 1.23 | 0.6319 | -0.66 | 0.0050 | 0.0119 |
| 50 | PI(18:0/20:4) | PI | PI-H | 885.5500 | 762.4575 | 1.09 | 0.5530 | -0.85 | 0.0032 | 0.0082 |
| 51 | TG(12:0/12:0/20:3) | TG | TG+NH4 | 762.6606 | 1108.9600 | 2.07 | 2.4587 | 1.30 | 0.0000 | 0.0004 |
| 52 | TG(12:0/14:0/14:0) | TG | TG+NH4 | 712.6450 | 1138.8850 | 1.50 | 2.3908 | 1.26 | 0.0000 | 0.0001 |
| 53 | TG(12:0/14:0/14:1) | TG | TG+NH4 | 710.6293 | 1097.0250 | 1.40 | 2.0351 | 1.03 | 0.0007 | 0.0028 |
| 54 | TG(12:0/16:1/18:3) | TG | TG+NH4 | 788.6761 | 1110.3200 | 1.73 | 3.1055 | 1.63 | 0.0000 | 0.0001 |
| 55 | TG(12:0/17:1/14:1) | TG | TG+NH4 | 750.6608 | 1123.3950 | 1.08 | 3.5906 | 1.84 | 0.0000 | 0.0000 |
| 56 | TG(13:0/15:1/17:0) | TG | TG+NH4 | 780.7076 | 1190.4550 | 1.10 | 1.7785 | 0.83 | 0.0011 | 0.0036 |
| 57 | TG(13:0/16:1/16:1) | TG | TG+NH4 | 778.6920 | 1157.9200 | 1.23 | 2.5854 | 1.37 | 0.0000 | 0.0002 |
| 58 | TG(14:0/14:0/14:0) | TG | TG+NH4 | 740.6764 | 1174.2550 | 1.25 | 1.6243 | 0.70 | 0.0006 | 0.0025 |
| 59 | TG(14:0/14:0/14:1) | TG | TG+NH4 | 738.6604 | 1138.8500 | 2.44 | 1.8802 | 0.91 | 0.0001 | 0.0005 |
| 60 | TG(14:0/16:0/16:0) | TG | TG+NH4 | 796.7390 | 1234.0850 | 1.87 | 0.3812 | -1.39 | 0.0260 | 0.0451 |
| 61 | TG(14:0/16:1/14:0) | TG | TG+NH4 | 766.6919 | 1173.7250 | 2.59 | 1.4788 | 0.56 | 0.0001 | 0.0009 |
| 62 | TG(14:0/18:2/18:2) | TG | TG+NH4 | 844.7387 | 1180.8400 | 4.38 | 1.5814 | 0.66 | 0.0017 | 0.0050 |
| 63 | TG(14:0/18:3/14:0) | TG | TG+NH4 | 790.6916 | 1142.6450 | 3.13 | 1.8841 | 0.91 | 0.0005 | 0.0023 |
| 64 | TG(14:0/18:3/16:1) | TG | TG+NH4 | 816.7073 | 1148.7850 | 3.39 | 2.2247 | 1.15 | 0.0001 | 0.0006 |
| 65 | TG(14:0/18:3/18:2) | TG | TG+NH4 | 842.7229 | 1152.4600 | 3.82 | 2.7506 | 1.46 | 0.0000 | 0.0001 |
| 66 | TG(14:0/18:3/18:3) | TG | TG+NH4 | 840.7063 | 1124.2850 | 1.73 | 6.4283 | 2.68 | 0.0000 | 0.0000 |
| 67 | TG(14:1/14:0/14:1) | TG | TG+NH4 | 736.6448 | 1103.1100 | 1.87 | 2.0735 | 1.05 | 0.0004 | 0.0019 |
| 68 | TG(14:1/18:3/16:1) | TG | TG+NH4 | 814.6919 | 1115.1500 | 1.55 | 4.0831 | 2.03 | 0.0000 | 0.0000 |
| 69 | TG(15:0/17:1/15:0) | TG | TG+NH4 | 808.7391 | 1218.4800 | 1.49 | 1.4427 | 0.53 | 0.0051 | 0.0119 |
| 70 | TG(15:1/15:0/17:2) | TG | TG+NH4 | 804.7078 | 1163.6800 | 1.40 | 3.0872 | 1.63 | 0.0000 | 0.0001 |
| 71 | TG(15:1/17:0/15:1) | TG | TG+NH4 | 806.7235 | 1191.1450 | 1.69 | 1.7284 | 0.79 | 0.0015 | 0.0047 |
| 72 | TG(15:1/17:1/17:1) | TG | TG+NH4 | 832.7390 | 1192.4300 | 2.44 | 2.0937 | 1.07 | 0.0002 | 0.0009 |
| 73 | TG(15:1/17:1/17:2) | TG | TG+NH4 | 830.7232 | 1166.4550 | 1.49 | 3.8241 | 1.94 | 0.0000 | 0.0001 |
| 74 | TG(15:1/18:2/18:2) | TG | TG+NH4 | 856.7391 | 1167.9700 | 1.44 | 4.0227 | 2.01 | 0.0000 | 0.0001 |
| 75 | TG(16:0/16:0/18:1) | TG | TG+NH4 | 850.7858 | 1258.1300 | 3.62 | 0.7632 | -0.39 | 0.0223 | 0.0397 |
| 76 | TG(16:0/16:1/16:1) | TG | TG+NH4 | 820.7387 | 1203.5300 | 2.70 | 0.8673 | -0.21 | 0.0058 | 0.0132 |
| 77 | TG(16:0/18:0/18:0) | TG | TG+NH4 | 880.8328 | 1299.3200 | 1.40 | 0.6935 | -0.53 | 0.0319 | 0.0535 |
| 78 | TG(16:0/18:0/18:1) | TG | TG+NH4 | 878.8171 | 1278.1200 | 5.04 | 1.4630 | 0.55 | 0.0039 | 0.0097 |
| 79 | TG(16:0/18:1/16:1) | TG | TG+NH4 | 848.7702 | 1229.3100 | 6.04 | 0.6574 | -0.61 | 0.0003 | 0.0015 |
| 80 | TG(16:0/18:1/17:0) | TG | TG+NH4 | 864.8015 | 1266.9750 | 3.57 | 1.8697 | 0.90 | 0.0000 | 0.0002 |
| 81 | TG(16:0/18:1/21:0) | TG | TG+NH4 | 920.8643 | 1305.4950 | 1.32 | 4.8679 | 2.28 | 0.0000 | 0.0001 |
| 82 | TG(16:0/18:3/18:2) | TG | TG+NH4 | 870.7543 | 1186.2150 | 6.08 | 1.8406 | 0.88 | 0.0002 | 0.0012 |
| 83 | TG(16:0/20:1/20:1) | TG | TG+NH4 | 932.8638 | 1294.4250 | 3.57 | 3.1323 | 1.65 | 0.0000 | 0.0001 |
| 84 | TG(16:0/20:4/18:1) | TG | TG+NH4 | 898.7856 | 1224.0700 | 2.04 | 1.3771 | 0.46 | 0.0056 | 0.0128 |
| 85 | TG(16:0/20:4/18:2) | TG | TG+NH4 | 896.7699 | 1198.4300 | 2.16 | 1.5722 | 0.65 | 0.0002 | 0.0012 |
| 86 | TG(16:0/22:4/18:1) | TG | TG+NH4 | 926.8167 | 1241.4900 | 1.85 | 1.4445 | 0.53 | 0.0022 | 0.0062 |
| 87 | TG(16:0/22:5/18:1) | TG | TG+NH4 | 924.8013 | 1221.9900 | 2.39 | 1.6232 | 0.70 | 0.0000 | 0.0002 |
| 88 | TG(16:0/22:5/18:2) | TG | TG+NH4 | 922.7854 | 1197.3400 | 1.99 | 1.9699 | 0.98 | 0.0000 | 0.0002 |
| 89 | TG(16:1/12:0/16:1) | TG | TG+NH4 | 764.6760 | 1141.7950 | 3.37 | 1.8407 | 0.88 | 0.0002 | 0.0013 |
| 90 | TG(16:1/14:0/16:1) | TG | TG+NH4 | 792.7074 | 1174.1900 | 2.68 | 1.2863 | 0.36 | 0.0139 | 0.0270 |
| 91 | TG(16:1/16:1/16:1) | TG | TG+NH4 | 818.7229 | 1176.1700 | 3.15 | 1.3240 | 0.40 | 0.0184 | 0.0341 |
| 92 | TG(16:1/16:1/17:0) | TG | TG+NH4 | 834.7546 | 1218.7950 | 2.37 | 1.4363 | 0.52 | 0.0050 | 0.0119 |
| 93 | TG(16:1/18:3/18:2) | TG | TG+NH4 | 868.7385 | 1155.0000 | 4.12 | 3.7220 | 1.90 | 0.0001 | 0.0005 |
| 94 | TG(16:1/18:3/18:3) | TG | TG+NH4 | 866.7188 | 1126.0700 | 1.32 | 8.1560 | 3.03 | 0.0000 | 0.0000 |
| 95 | TG(17:0/15:0/17:1) | TG | TG+NH4 | 836.7702 | 1244.3450 | 2.70 | 1.5266 | 0.61 | 0.0004 | 0.0017 |
| 96 | TG(17:0/18:0/18:1) | TG | TG+NH4 | 892.8328 | 1287.2600 | 2.86 | 3.2500 | 1.70 | 0.0000 | 0.0001 |
| 97 | TG(17:0/18:1/18:1) | TG | TG+NH4 | 890.8168 | 1266.1400 | 3.12 | 1.6486 | 0.72 | 0.0006 | 0.0025 |
| 98 | TG(17:0/18:2/20:1) | TG | TG+NH4 | 916.8327 | 1266.1700 | 1.47 | 1.8178 | 0.86 | 0.0010 | 0.0034 |
| 99 | TG(17:1/17:1/17:1) | TG | TG+NH4 | 860.7703 | 1219.7450 | 3.05 | 1.6430 | 0.72 | 0.0022 | 0.0062 |
| 100 | TG(17:1/17:1/17:2) | TG | TG+NH4 | 858.7546 | 1194.7600 | 2.76 | 2.5439 | 1.35 | 0.0002 | 0.0010 |
| 101 | TG(17:1/17:1/21:0) | TG | TG+NH4 | 918.8485 | 1286.2900 | 1.92 | 3.2532 | 1.70 | 0.0000 | 0.0001 |
| 102 | TG(17:1/18:2/18:1) | TG | TG+NH4 | 886.7857 | 1220.5400 | 2.48 | 1.9863 | 0.99 | 0.0029 | 0.0076 |
| 103 | TG(18:0/18:0/18:1) | TG | TG+NH4 | 906.8483 | 1297.2750 | 5.28 | 2.8380 | 1.50 | 0.0000 | 0.0002 |
| 104 | TG(18:0/18:1/20:0) | TG | TG+NH4 | 934.8800 | 1314.7700 | 2.51 | 7.1323 | 2.83 | 0.0007 | 0.0027 |
| 105 | TG(18:0/18:1/22:0) | TG | TG+NH4 | 962.9116 | 1331.2950 | 1.43 | 8.4140 | 3.07 | 0.0000 | 0.0000 |
| 106 | TG(18:1/18:0/18:1) | TG | TG+NH4 | 904.8322 | 1275.2950 | 3.50 | 1.3174 | 0.40 | 0.0122 | 0.0241 |
| 107 | TG(18:1/18:1/20:1) | TG | TG+NH4 | 930.8482 | 1273.3750 | 1.93 | 1.6604 | 0.73 | 0.0017 | 0.0050 |
| 108 | TG(18:1/18:1/22:0) | TG | TG+NH4 | 960.8956 | 1312.8850 | 2.16 | 5.9534 | 2.57 | 0.0000 | 0.0000 |
| 109 | TG(18:1/18:1/22:1) | TG | TG+NH4 | 958.8796 | 1293.2700 | 1.65 | 3.1442 | 1.65 | 0.0000 | 0.0001 |
| 110 | TG(18:2/17:1/18:2) | TG | TG+NH4 | 884.7701 | 1195.7050 | 1.98 | 2.6795 | 1.42 | 0.0007 | 0.0027 |
| 111 | TG(18:2/18:2/19:0) | TG | TG+NH4 | 914.8171 | 1245.5300 | 1.09 | 1.8883 | 0.92 | 0.0018 | 0.0051 |
| 112 | TG(18:2/18:3/18:2) | TG | TG+NH4 | 894.7531 | 1158.1600 | 3.68 | 3.6664 | 1.87 | 0.0001 | 0.0006 |
| 113 | TG(18:2/18:3/18:3) | TG | TG+NH4 | 892.7334 | 1129.5650 | 1.52 | 10.5840 | 3.40 | 0.0000 | 0.0000 |
| 114 | TG(20:0/20:1/20:1) | TG | TG+NH4 | 988.9273 | 1329.7350 | 1.40 | 9.5051 | 3.25 | 0.0000 | 0.0000 |
| 115 | TG(20:1/17:0/20:1) | TG | TG+NH4 | 946.8801 | 1304.5250 | 1.06 | 4.7927 | 2.26 | 0.0000 | 0.0001 |
| 116 | TG(22:1/16:0/22:2) | TG | TG+NH4 | 986.9114 | 1311.9300 | 1.43 | 5.8963 | 2.56 | 0.0000 | 0.0000 |

Rt, retention time; VIP, variable importance in projection; FC, Fold change; FDR, false discovery rate. Cer, Ceramides; LPC, Lysophosphatidylcholine; LPE, Lysophosphatidylethanolamine; PA, Phosphatidic acid; PC, Phosphatidylcholine; PE, Phosphatidylethanolamine; PI, Phosphatidylinositol; TG, Triglyceride.

**Table S4 Receiver operating characteristic parameter of different lipids in cook and raw of donkey muscles**

| No. | Lipid name | AUC | Ci1 | Ci2 | Specificity  (%) | Sensitivity  (%) | Threshold |
| --- | --- | --- | --- | --- | --- | --- | --- |
| 1 | PC(O-16:2/2:0) | 1 | 1 | 1 | 1 | 1 | 102287885.34 |
| 2 | LPE(22:5/0:0) | 1 | 1 | 1 | 1 | 1 | 5586475.45 |
| 3 | PC(P-16:0/2:0) | 1 | 1 | 1 | 1 | 1 | 15087746.73 |
| 4 | TG(18:2/18:3/18:3) | 1 | 1 | 1 | 1 | 1 | 5307451.32 |
| 5 | TG(20:0/20:1/20:1) | 1 | 1 | 1 | 1 | 1 | 4120493.84 |
| 6 | TG(18:0/18:1/22:0) | 1 | 1 | 1 | 1 | 1 | 4380439.52 |
| 7 | TG(16:1/18:3/18:3) | 1 | 1 | 1 | 1 | 1 | 4870316.03 |
| 8 | TG(18:0/18:1/20:0) | 1 | 1 | 1 | 1 | 1 | 13749764.78 |
| 9 | TG(14:0/18:3/18:3) | 1 | 1 | 1 | 1 | 1 | 8673840.19 |
| 10 | TG(18:1/18:1/22:0) | 1 | 1 | 1 | 1 | 1 | 12897316.92 |
| 11 | TG(22:1/16:0/22:2) | 1 | 1 | 1 | 1 | 1 | 5006386.48 |
| 12 | TG(16:0/18:1/21:0) | 1 | 1 | 1 | 1 | 1 | 4763494.71 |
| 13 | TG(20:1/17:0/20:1) | 1 | 1 | 1 | 1 | 1 | 3176893.00 |

Area under the receiver operating characteristic (ROC) curve (AUC) was the area under ROC curve, Ci1 is the lower limit of AUC confidence interval; Ci2 is the upper limit of AUC confidence interval. PC, Phosphatidylcholine; LPE, Lysophosphatidylethanolamine; TG, Triglyceride.
